# Human Vγ9Vδ2 T Lymphocytes in the Immune Response to *P. falciparum* Infection

**DOI:** 10.3389/fimmu.2018.02760

**Published:** 2018-11-27

**Authors:** Jennifer Howard, Irfan Zaidi, Séverine Loizon, Odile Mercereau-Puijalon, Julie Déchanet-Merville, Maria Mamani-Matsuda

**Affiliations:** ^1^Division of Intramural Research (DIR), National Institutes of Allergy and Infectious Diseases, Bethesda, MD, United States; ^2^Univ. Bordeaux, CNRS ImmunoConcEpT UMR 5164, Bordeaux, France; ^3^Parasites and Insect Vectors Department, Institut Pasteur, Paris, France

**Keywords:** gamma-delta T cells, malaria, falciparum, immunity to malaria, antigen presenting cell (APC), cytotoxicity

## Abstract

Malaria is an infectious disease caused by the protozoan parasite *Plasmodium sp*, the most lethal being *Plasmodium falciparum*. Clinical malaria is associated with the asexual replication cycle of *Plasmodium* parasites inside the red blood cells (RBCs) and a dysregulated immune response. Although the mechanisms of immune responses to blood—or liver-stage parasites have been extensively studied, this has not led to satisfactory leads for vaccine design. Among innate immune cells responding to infection are the non-conventional gamma-delta T-cells. The Vγ9Vδ2 T-cell subset, found only in primates, is activated in response to non-peptidic phosphoantigens produced by stressed mammalian cells or by microorganisms such as Mycobacteria, *E*.*coli*, and *Plasmodium*. The potential protective role of Vγ9Vδ2 T-cells against infections and cancer progression is of current research interest. Vγ9Vδ2 T-cells have been shown to play a role in the early control of *P. falciparum* parasitemia and to influence malaria adaptive immunity *via* cytokine release and antigen presentation. They are activated and expanded during a primary *P. falciparum* infection in response to malaria phosphoantigens and their activity is modulated upon subsequent infections. Here, we review the wide range of functions by which Vγ9Vδ2 T-cells could both contribute to and protect from malaria pathology, with a particular focus on their ability to induce both innate and adaptive responses. We discuss how the multifunctional roles of these T-cells could open new perspectives on gamma-delta T-cell-based interventions to prevent or cure malaria.

## Introduction

Over the last decades, the importance of a specific subset of γδ T-cells in malaria infection is becoming increasingly apparent, namely Vγ9Vδ2 T-cells. Restricted to human and non-human primates, Vγ9Vδ2 T-cells constitute a non-conventional T-cell subset activated in a non-MHC dependent manner, by phosphorylated intermediates of isoprenoid biosynthesis pathways of mammalian cells and microorganisms, known as phospho-antigens (Ph-Ag) (1). The known most potent of these, HMBPP [(E)-4-Hydroxy-3-methyl-but-2-enyl pyrophosphate] is produced by the DOX-P pathway used by several microorganisms (2) including the parasite responsible for malaria, *Plasmodium spp* [reviewed (3)]. Once activated, Vγ9Vδ2 T-cells expand, produce cytokines, exert cytotoxic functions, and stimulate cells such as monocytes, resulting in improved monocyte antigen presentation capabilities (4).

Despite major global effort, malaria remains a major public health concern. Nearly half of the world's population live in malaria endemic regions, the majority in sub-saharan Africa, and it is responsible for ~216 million cases and 445,000 deaths each year (5)**. **Efforts to create an effective vaccine are hampered by lack of understanding of the parasites interactions with our immune system.

There are five species of *Plasmodium* that infect humans: *P. falciparum, P. vivax, P. ovale, P. malariae*, and *P. knowelsi*. *P. falciparum* is the most prevalent and deadly. *P. falciparum*, similar to other *Plasmodium*, is transmitted through the bite of a female *Anopheles* mosquito. The extracellular, liver-invasive form, the sporozoite, is injected into the skin, where it enters the blood flow and travels to the liver. Here the parasite eventually invades hepatocytes, wherein it differentiates and divides to form the extracellular form called merozoites. Merozoites are released into the blood stream and invade red blood cells (RBCs) where they progress through a 48 h life cycle before RBC rupture and merozoite release. Clinical disease manifests during this blood stage and is characterized by cyclical episodes of fever paroxysms. Severe malaria can be fatal and presents an array of severe symptoms including severe anemia, respiratory distress caused by severe metabolic acidosis, cerebral-malaria, multi-organ failure, and in pregnant women, placental malaria (6).

For over 100 years, it has been observed that partial immunity to malaria in endemic areas is only acquired after multiple disease episodes (7–9). In endemic settings, immunity is developed first to severe malaria (usually before 5 years old) then to clinical malaria (by 10–15 years old) (8, 10–12). Acquired immunity appears to be strain- and variant-specific and in endemic areas people are frequently re-infected by novel variants with novel antigen combinations. This complicates the assessment of protective immunity, however it is commonly accepted that sterile immunity is rarely reached and low parasitemia with no clinical symptoms is instead maintained (13, 14). Malaria infection causes dysregulation of immune responses, including inhibition of DC maturation and antigen presenting capacity (15–17) and expansion of atypical memory B cells, the functionality of which is not yet understood (18–20). The role of the innate immune responses, and the cellular and humoral branches of the adaptive immune response has been excellently reviewed elsewhere (11, 21–25).

Concerning γδ T-cells, much of the early *in vivo* work on Vγ9Vδ2 T-cell responses to *P. falciparum* infection was done in primary infected adult patients, usually Caucasians living in non-endemic regions, where Vγ9Vδ2 T-cells are the dominant subset of γδ T-cells. However, it has been shown that in malaria endemic regions, where the populations are exposed to numerous malaria infections and possibly chronically infected, Vδ1 T-cells are the major subset (26, 27). It is not yet known if this is a genetic peculiarity, or different microbiota and pathogen exposure early in life that drives expansion and contraction of these subsets. An in-depth discussion on the reasons for these geographical differences, and the role played by non Vγ9Vδ2 T-cells in malaria infection is beyond the scope of this review, which focuses on Vγ9Vδ2 T-cells. Vγ9Vδ2 T-cells have features associated with both innate and adaptive T-cells, and increasing evidence suggests they act as a bridge between the innate and adaptive immune systems [reviewed (28–30)]. Vγ9Vδ2 T-cells have a wide range of effector functions [reviewed (31, 30)], and it is becoming increasingly clear that during *P. falciparum* infection they contribute to both protection and pathology. In this review, we discuss their role as cytotoxic killer cells and their ability to initiate both innate and adaptive immune responses against *P. falciparum* malaria infection via cytokine release and direct antigen presentation to CD4 and CD8 T-cells.

## Vγ9vδ2 t-cells are activated during malaria infection

γδ T-cells have long been observed to expand *in vivo* in the peripheral blood of primary infected *P. falciparum* malaria patients, with the major subset being Vγ9Vδ2 T-cells (32, 33). Interestingly, expansion in the peripheral blood is greatest during recovery, after acute infection has passed (34), indicating either a delay in response, or homing to tissues during acute infection. Vγ9Vδ2 T-cells were found to be increased in human spleens during infection (35, 36), a phenomenon that was confirmed in monkey models (36). The rapid expansion of Vγ9Vδ2 T-cells during infection and their homing to sites of known importance in parasite clearance indicated that Vγ9Vδ2 T-cells could play a role in the response to infection.

Our previous work has demonstrated that the bioactive molecule released by infected red blood cells (iRBC) is a Ph-Ag of the DOX-P pathway, which is released concomitantly with iRBC rupture. We also showed that presentation of parasite Ph-Ag to Vγ9Vδ2 T-cells involves BNT3A1 on non-erythrocyte bystander cells, as RBCs and iRBCs are devoid of BNT3A1 (37). In addition to HMBPP various other signals, including IL-2, IL-15 (38, 39), CD4 T-cell interaction activation (40) and CD28 co-stimulation (41), are needed for effective Vγ9Vδ2 T-cell activation, and stimulation of Vγ9Vδ2 T-cells in different cytokine milieus emphasizes different functional behaviors (42).

## Cytotoxic vγ9vδ2 t-cells directly target blood stage *p. falciparum*

*In vitro* studies have built a picture of how Vγ9Vδ2 T-cells directly inhibit the erythrocyte stage life-cycle. The first studies showed that Vγ9Vδ2 T-cells targeted the iRBCs in a contact dependent manner, and suggested that merozoites were the target, as inhibition of parasite life-cycle was not seen until after parasite reinvasion (43–45). Active granulysin release by the Vγ9Vδ2 T-cells was implied in mediating parasite growth inhibition, as granulysin production correlated with life-cycle inhibition (44). Experiments with granulysin and perforin deficient Vγ9Vδ2 T-cell lines confirmed that Vγ9Vδ2 T-cell inhibition of parasites was indeed granulysin-mediated but not perforin-dependent (46). Finally, in an experiment where Vγ9Vδ2 T-cells were co-cultured with late stage iRBC and removed before rupture there was no impact on the parasite reinvasion. This showed definitively that merozoites are the target, as schizonts are not affected by granulysin release (46).

## Cytokine releasing vγ9vδ2 t-cells act as a trigger for both innate and adaptive immune responses

Vγ9Vδ2 T-cells are highly interactive, and much of their impact on the course of an immune response stems from their modulation of other innate and adaptive immune cells by cytokine release and direct cell-cell interaction (30). Existing evidence indicates that Vγ9Vδ2 T-cells are implicated in impacting the scale and nature of both innate and adaptive immune responses to *P. falciparum* infection. A large feature of the immune response to *P. falciparum* infection is the production of inflammatory cytokines. *In vitro* studies of schizont-activated PBMCs from naïve donors, Vγ9Vδ2 T-cells have been found to produce TNFα and be the major source of IFNγ, more than NK cells or macrophages (47–50). They have also been shown to express TNFα, TGF-β, and IL-8, and occasionally IL-10, IL-2, and IL-5 (48). In *ex vivo* analysis of cord blood from mothers in an endemic setting who had experienced malaria during pregnancy, the Vγ9Vδ2 T-cells produced significantly more IFNγ and TNFα than those from healthy mothers, as did the peripheral Vγ9Vδ2 T-cells from the mother (51). This inflammatory cytokine production by Vγ9Vδ2 T-cells has been associated with both protection and pathogenesis.

Vaccination studies have been performed where healthy, malaria naïve, volunteers are exposed to three doses of *P. falciparum* (via the bite of 12–15 infected mosquitos) with the accompaniment of chloroquine treatment. This permits the parasite to mature to blood stage, when it is then swiftly killed before disease symptoms can develop. After challenge by the bites of five infected mosquitos, the inoculated volunteers remained parasite-free, indicating that they had developed a sterilizing immunity (52, 53). Vaccinated (protected) volunteers showed increased IFNγ, TNFα, and IL-2 production compared to non-vaccinated (non-protected) when PBMCs, taken pre-challenge and 1 day post-challenge, were stimulated by iRBC *in vitro* (52, 53). IFNγ levels were also increased in PBMCs from vaccinated volunteers taken days 9, 35, 140, and 400 post-challenge when stimulated by both iRBC and sporozoites (53). γδ T-cells were found to be the major IFNγ contributors, with αβ T-cells the next largest. The majority of responding cells were effector memory, indicating recall responses, and IFNγ-producing γδ T-cells were demonstrated to be a major contributor to parasite-specific recall responses (53). Thus, in these vaccines, IFNγ production by lymphocytes including γδ T-cells, correlated with acquired immunity to *P. falciparum* infection. It should be noted that Vγ9Vδ2 T-cells were not specifically measured in this study. However, as Vγ9Vδ2T-cells are the predominant subset in the periphery of malaria naïve individuals from non-malaria endemic regions, it is reasonable to assume they were the major responding γδ T-cell subset in this study.

In longitudinal studies of semi-immune children from Papua New Guinea, the *in vitro* response of PMBCs to iRBC was measured, and subsequent malaria incidence recorded. Increased IFNγ production by PBMCs correlated with reduced risk of future moderate and high-density *P. falciparum* infection. Further, though there was much donor heterogeneity, γδ T-cells were the predominant IFNγ producing cell population (54). However, a different longitudinal study of children from Papua New Guinea suggests that γδ T-cell cytokine production is involved in severe malaria. *Ex vivo* stimulation of PBMCs from children with either severe or uncomplicated malaria or healthy controls showed that γδ T-cells and monocytes were responsible for inflammatory cytokines associated with ‘high odds' of severe malaria (55). Several studies together have shown that Vγ9Vδ2 T-cell cytokine production is abrogated with repeat malaria exposure, and this contributes to decreasing clinical symptoms in subsequent infections.

Decreased peripheral activity of Vγ9Vδ2 T-cells has been found during the acute stage of infection in primary *P. falciparum* infected adults. Vγ9Vδ2 T-cells taken from the peripheral blood during paroxysms were found to expand less and produce less TNFα in response to IPP stimulation than Vγ9Vδ2 T-cells taken during recovery, post-treatment (though still expanded compared to uninfected controls). It was also found that there are less Vγ9Vδ2 T-cells [particularly Vγ2Jγ1.2γδ T-cells (US nomenclature), the TCR subset that is particularly reactive to Ph-Ags] in circulation during *P. falciparum* paroxysm than during recovery (34).

In a longitudinal study of Ugandan children, the percentage of Vγ9Vδ2 T-cells in peripheral blood was found to be inversely correlated with prior incidence of malaria infections. *Ex vivo*, Vγ9Vδ2 T-cell proliferation, TNFα, and IFNγ production and immune-modulatory gene expression was also negatively associated with prior malaria episodes—indicating decreased peripheral blood Vγ9Vδ2 T-cell activity with increasing exposure to the parasite. Lower *in vitro* Vγ9Vδ2 T-cell responsiveness to iRBC correlated with lower subsequent incidences of symptomatic infection, but to increased probability of higher parasitemia (56). This Vγ9Vδ2 T-cell dysfunction was shown to occur because of frequent malaria episodes in childhood, an effect that was abrogated by chemoprevention in early childhood (57). The mechanism of Vγ9Vδ2 T-cell regulation is as yet unknown. Vγ9Vδ2 T-cells are very susceptible to activation-induced cell death by Fas-Fas-L interaction as demonstrated for *M. tuberculosis* (58), though active regulation cannot be ruled out.

Together, these studies indicate that while Vγ9Vδ2 T-cell inflammatory cytokine responses can control parasitemia, excessive stimulation of these cells may also result in pathology suggesting that clinical immunity to malaria may be associated with reduced Vγ9Vδ2 responses.

Several accumulated data in mice, where the equivalent of human Vγ9Vδ2 T-cell subset is not yet certain, also show the importance of the cytokine secretion activity of murine γδ T-cells (59, 60). A recent study (61) showed that clonal expansion of a subset of γδ T-cells producing macrophage colony stimulating factor (M-CSF), prevents parasitemic recurrence. While it is perhaps a stretch to expect a direct murine equivalent of Vγ9Vδ2 T-cells, certainly one is not yet identified, it is likely that one or more murine γδ T-cell subsets have evolved which perform the same protective and/or pathologic functions in malaria infection as human Vγ9Vδ2 T-cells. “TγδM” cells are a good candidate for one such functional equivalent of Vγ9Vδ2 T-cells.

## Antigen presenting vγ9vδ2 t-cells stimulate adaptive immune responses

Another way in which Vγ9Vδ2 T-cells influence the course of an immune response is by antigen presentation to αβ T-cells. Over the last 12 years it has been demonstrated that Vγ9Vδ2 T-cells can take up, process and present exogenous Ag, both via the classical pathway to CD4 T-cells and the cross-presentation pathway to CD8 T-cells. They even have shown the ability to act as professional antigen presenting cells (APCs) and stimulate naive CD4 and CD8 T-cells (62–70). γδ T-APC resembling cells are present in malaria infected individuals, and *in vitro* iRBC stimulated Vγ9Vδ2 T-cells not only take on an APC phenotype but also can cross-present Ag to a memory cell line and activate naïve CD4 and CD8 T-cells in a mixed-lymphocyte reaction (71). Where this might be occurring *in vivo*, or what the implication of repeated malaria infection could be is worth investigated. However, interesting work from liver stage malaria vaccines could shed some light on this (see below).

**Figure 1:**
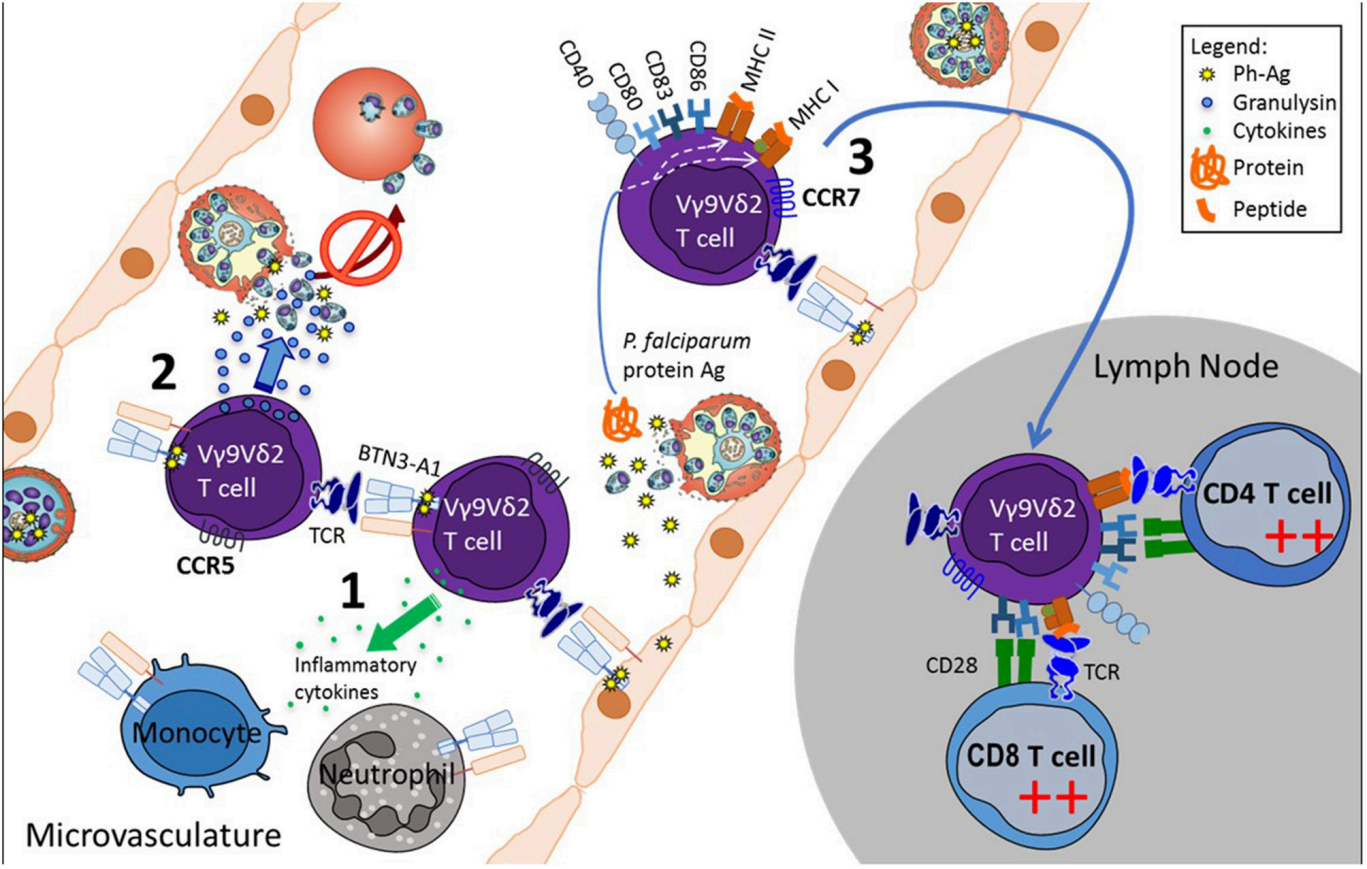
Proposed model of Vγ9Vδ2 T-cell functions in the microvasculature during *P. falciparum* infection. Plasmodium-infected red blood cells sequester to the endothelium in the microvasculature, where they release phosphoantigens concomitantly with the red blood cell rupture. Phosphoantigens stimulate Vγ9Vδ2 T-cells via BTN3A1 available on neighboring cells, including Vγ9Vδ2 T-cells, the endothelial cells and innate immune cells. Activated Vγ9Vδ2 T-cells (1) modulate innate cells by cytokine secretion, (2) inhibit free parasite reinvasion of red blood cells, by releasing the cytotoxic granulysin, and (3) acquire APC phenotype and the capability to migrate to lymph nodes where they initiate an adaptive immune response.

Overall, the data allow us to propose a global model of how peripheral Vγ9Vδ2 T-cells could control parasitemia and initiate both innate and adaptive responses (Figure 1). Whether the same cells are responsible for these functions or whether different subsets of Vγ9Vδ2 T-cell are concerned is still to be worked out.

## Vγ9vδ2 t-cells: correlates of protection for whole organism malaria vaccine?

Vγ9Vδ2 T-cells have been implicated in protection against liver stage immunity after vaccination with whole sporozoites.

In a mouse model of irradiated sporozoite vaccinations, it was clear that the γδ T-cells were required for induction of protective CD8 T-cell responses, but not antibodies, and were not acting as effectors in controlling liver stage parasite replication (72). An as yet undefined subset of mouse γδ T-cells are able to function by inducing downstream αβ T-cell responses. Further studies are required to establish which mouse γδ T-cell subsets mirror the various activities of Vγ9Vδ2 γδ T-cells and explore the effect of irradiated sporozoite vaccination dose on these cells. In humans, in the first field trial of the Sanaria® PfSPZ vaccine in Mali, it was demonstrated that the Vγ9Vδ2 T-cells were highest in vaccines that remained uninfected throughout an intense malaria transmission season, compared to infected vaccines or the placebo group (72). These findings were comparable to those observed in malaria naïve individuals vaccinated with either the PfSPZ vaccine or a chemoprophylaxis vaccination, who also had a remarkable increase in Vγ9Vδ2 T-cells (73). Overall, these findings are intriguing in that they suggest that liver-stages growth of *P. falciparum* can stimulate Vγ9Vδ2 T-cell activation. This activation could have several explanations: first, locally in an infected liver, hepatocytes displaying BNT3A1 or other presentation molecules could activate Vγ9Vδ2 T-cells *in situ*. Second, Vγ9Vδ2 T-cells could be activated in the draining lymph nodes of the site of infection where a substantial fraction of the sporozoites migrate, as shown by Amino et al. in mouse model (74). Third, HMBPP produced by liver stages of *Plasmodium* could be sensed in the periphery by exquisitely sensitive Vγ9Vδ2 T-cells, as seen during blood stage *P. falciparum* infections (37). Finally, the activation of Vγ9Vδ2 T-cells could be due to recognition of other antigens or metabolites.

It should be noted that in subsequent trials which used a higher dose of the PfSPZ vaccine, Vγ9Vδ2 T-cell expansion did not distinguish protected vs. unprotected vaccines (75, 76). Interestingly, liver stage induced Vγ9Vδ2 T-cell expansion has not been observed in volunteers undergoing controlled human malaria infections (77). The reasons behind this are not yet understood, but given the plasticity of Vγ9Vδ2 T-cells, it may be that varying antigen loads modulate the phenotype and function of these cells.

## Concluding remarks

In conclusion, the Vγ9Vδ2 T-cell is an enigmatic cell, with a wide range of functions that can both contribute to and protect from malaria pathology. It is important to better consider this subset of γδ T-cells, especially their role in malaria vaccine protection. Given their sensitivity to Ph-Ag's such as HMBPP and apparent functional plasticity under different cytokines and stimuli dose, a cocktail of Ph-Ag and cytokines could be envisioned as an adjuvant to boost efficacy of both liver and blood stage malaria vaccines.

## Author contributions

JH, IZ, and MM-M wrote the manuscript. SL and OM-P contributed to the manuscript. JD-M secured fundings.

## Conflict of interest statement

The authors declare that the research was conducted in the absence of any commercial or financial relationships that could be construed as a potential conflict of interest.
